# Feasibility of establishing a core set of sexual, reproductive, maternal, newborn, child, and adolescent health indicators in humanitarian settings: results from a multi-methods assessment in Bangladesh

**DOI:** 10.1186/s12978-022-01424-8

**Published:** 2022-05-21

**Authors:** Bachera Aktar, Kanya Lakshmi Rajendra, Emily Clark, Kassandre Messier, Anya Aissaoui, Kaeshan Elamurugan, Md. Tanvir Hasan, Nadia Farnaz, Adrita Kaiser, Abdul Awal, Ieman Mona El Mowafi, Loulou Kobeissi

**Affiliations:** 1 BRAC James P. Grant School of Public Health, BRAC University, Dhaka, Bangladesh; 2 Department of Health Behavior and Health Education, School of Public Health, University of Michigan, Ann Arbor, MI USA; 3 NOR Impact AS, Rogaland, Norway; 4 Faculty of Health Sciences, University of Ottawa, Ottawa, ON Canada; 5 Cambridge Reproductive Health Consultants, Cambridge, MA USA; 6 Institute for Population Health, University of Ottawa, Ottawa, ON Canada; 7 Department of Sexual and Reproductive Health and Research (SRH), World Health Organization, Geneva, Switzerland; 8 New Mexico Institute of Mining and Technology, Socorro, NM USA

**Keywords:** Bangladesh, Monitoring and Evaluation Sexual and Reproductive Health, Maternal, Child and Adolescent Health, Humanitarian data reporting, Health Information Systems, Refugees, Refugee Health

## Abstract

**Background:**

Reliable and rigorously collected sexual, reproductive, maternal, newborn, child, and adolescent health (SRMNCAH) data in humanitarian settings is often sparse and varies in quality across different humanitarian settings. To address this gap in quality data, the World Health Organization (WHO) developed a core set of indicators for monitoring and evaluating SRMNCAH services and outcomes, and assessed their feasibility in Bangladesh, Afghanistan, Jordan, and the Democratic Republic of Congo.

**Methods:**

The feasibility assessments aggregated information from global consultations and field-level assessments to reach a consensus on a set of core SRMNCAH indicators among WHO partners. The feasibility assessment in Bangladesh focused on the following constructs: relevance/usefulness of the core set of indicators, the feasibility of measurement, availability of systems and resources, and ethical issues during data collection and management. The field-level multi-methods assessment included five components; a desk review, key informant interviews, focus group discussions, and facility assessments including observations of facility-level data management.

**Results:**

The findings suggest that there is widespread support among stakeholders for developing a standardized core set of SRMNCAH indicators to be collected among all humanitarian actors in Bangladesh. There are numerous resources and data collection systems that could be leveraged, built upon, and improved to ensure the feasibility of collecting this proposed set of indicators. However, the data collection load requested from donors, the national government, international and UN agencies, coordination/cluster systems must be better harmonized, standardized, and less burdensome.

**Conclusion:**

This core set of indicators would only be useful if it has the buy-in from the international community that results in harmonizing and coordinating data collection efforts and relevant indicators’ reporting requirements.

## Plain English Summary

Reliable data on sexual, reproductive, maternal, newborn, child and adolescent health (SRMNCAH) in humanitarian settings is very important for addressing the needs of the refugee population. However, the quality of data collected often varies across different humanitarian settings and organizations. To address this gap, WHO developed a core set of indicators, through global consultations and field-level assessments, for monitoring and evaluating SRMNCAH services and outcomes in humanitarian settings. In Bangladesh, the feasibility assessment was conducted in Rohingya camps in Cox’s Bazar and assessed the feasibility in terms of relevance/usefulness of the indicators, feasibility of measurement, availability of systems and resources for data collection, and data collection and management related ethical issues in the Rohingya context in Bangladesh. The field-level assessment applied a multi-method approach including a desk review, key informant interviews, focus group discussions, and facility assessments. The findings revealed existing multiple sources and vertical systems of data collection by different organizations and the indicators also varied depending on the requirements of the Bangladesh government, donor agencies, UN agencies leading different sector/sub-sectors/clusters, and organizations’ own priorities and mandates. All the stakeholders who participated in this study agreed on developing a harmonized and standardized core set of SRMNCAH indicators. However, they raised concerns regarding the adaptation of the core set of indications to the local context. They also emphasized on the importance of adequate resources for establishing strong reporting and data management systems, capacity development of human resources and the buy-in from the international community for effective implementation.

## Introduction

### The Rohingya humanitarian crisis in Cox’s Bazar, Bangladesh

Since the early 1990s, the Rohingya population have been experiencing discrimination and communal violence in Myanmar and seeking refuge in neighboring countries, including Bangladesh [1]. Tensions between the Rohingya and the predominantly Buddhist population of Myanmar, have continued to rise over the past few decades with subsequent bursts of conflict and violence [2]. The latest conflict that happened in August 2017 led to the largest mass displacement of Rohingyas in history, with the most recent UNHCR estimates suggesting that more than 900,000 people were displaced [3]. The vast majority of Rohingya diaspora or forcibly displaced Myanmar nationals (FDMN)^1^ are camp-based and reside within two sub-districts of Cox’s Bazar district [4]. More than half of FDMNs are women and girls, and an estimated 22,000 women are pregnant at any given point in time [3, 5]. Although considerable efforts have been made by the Government of Bangladesh and different humanitarian actors to stabilize the situation, the Rohingyas continue to face considerable challenges in accessing comprehensive sexual, reproductive, maternal, newborn, child, and adolescent health (SRMNCAH) care.

### SRMNCAH data collection and indicator reporting

Consistently cited in the literature is the need for robust, reliable, and timely information and data to identify and respond with evidence-based interventions for complex humanitarian crisis conditions and their affected populations [6–8]. As forced displacement increases the health risk of the affected population, relevant context-specific public health information is crucial to support and enable the local health system^2^ to respond to the crisis situation [10, 11]. Accurate and reliable health information enables humanitarian aid organizations to invest and prioritize resources effectively and efficiently [7, 12]. Implementing a system for reliable and rigorous data collection, aggregation, and use, would allow organizations and agencies providing sexual, reproductive, maternal, newborn, child, and adolescent health (SRMNCAH) services to accurately and consistently report on their programmatic activities [12, 13]. This, then, allows for responsive and evidence-based decision making on policy, funding, program development, and implementation. Further, establishing this system would improve the accountability of humanitarian actors in providing SRMNCAH services to vulnerable populations in humanitarian settings [13]. Developing a core set of indicators to collect SRMNCAH related data across agencies and organizations, would allow accurate tracking of inputs, processes, outcomes, and impact within a set context.

### SRMNCAH data collection and indicator reporting in Cox’s Bazar

Throughout the ongoing humanitarian crisis in Cox’s Bazar there have been continuous efforts from the Government of Bangladesh and aid agencies to try and improve the monitoring of health services in the region [14]. FDMNs receive health services through various providers from the Ministry of Health and Family Welfare of the Government of Bangladesh, UN agencies, and NGOs [15]. Earlier work documented that there was insufficient distribution and duplication of services in the camps [13, 16]. This then led the health sector to initiate a rationalization process to integrate, relocate, and upgrade health facilities and services [14, 17].

There are several Health Information Systems (HIS) and tools currently in place to monitor and evaluate health status and the delivery and use of health services, and these vary depending on the organization or agency in question. The Government of Bangladesh employs the District Health Information System version 2 (DHIS2) for its monitoring and evaluation practices [17] and requires many aid agencies to report to this system as well. In addition to this, many agencies employ several other reporting systems for internal use and/or for donor requirements. This patchwork of reporting systems and requirements leads to duplicate, untimely, and incomplete data, as well as poses an unnecessary burden on staff [14, 16, 18].

In light of the above, WHO, in close coordination with national, regional and global partners, took the initiative to assess the feasibility, relevance, and acceptability of a core set of SRMNCAH indicators for humanitarian settings in four countries experiencing different types of humanitarian crises including the Rohingya crisis in Bangladesh. In Bangladesh, the assessment took place in camp-based settings in Cox’s Bazar. This paper discusses the results obtained from these assessment in Bangladesh.

By assessing feasibility, we aimed to explore the feasibility of the proposed SRMNCAH framework, whether or not national and non-governmental monitoring and evaluation systems have the needed resources to collect SRMNCAH indicators, and the ability of the system to adhere to ethical practice and safeguard clients’ confidentiality and privacy. The results of Bangladesh’s country level feasibility assessment were aggregated with the results from Afghanistan, Jordan, and Democratic Republic of Congo’s field-level assessments in order to reach a global consensus on a minimum set of core SRMNCAH indicators for services and outcomes monitoring and evaluation in humanitarian settings among donor agencies, UN agencies, and international NGOs working in humanitarian settings.

## Methods

### Study design

**Fig. 1:**
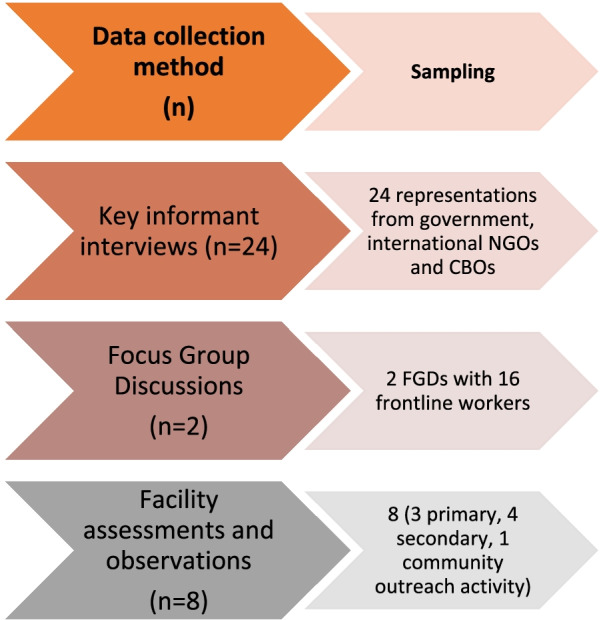
Data collection for Phase II. NGO: Non-governmental organization; CBO: Community based organization

Rogers’ diffusion of innovations theory undergirds this project [19], we hypothesize that the adoption and diffusion of a core set of SRMNCAH indicators will involve a stage-based progression: *awareness* of the need for a new intervention; *decision to adopt* (or reject) the new intervention; and *continued use* of the new intervention. As such, we focused our feasibility assessment on the following constructs: relevance/usefulness, feasibility of measurement, systems and resources, and ethical issues. This study used a multi-methods assessment consisting of five main components: (1) a desk review of published articles and reports as well as internal documents (in English and Bengali); (2) key informant interviews (KIIs) with representatives from government entities, national and international non-governmental organizations (NGOs); (3) facility assessments at primary, secondary, and community outreach healthcare facilities that provide services to refugees in Cox’s Bazar, Bangladesh; (4) observation sessions focused on the logistical, ethical, and privacy practices associated with data collection and storage at selected facilities; and (5) focus group discussions (FGDs) with frontline workers and their supervisors at primary, secondary, and community outreach health centers (see Fig. 1).

The assessment centered on seeking and understanding the different stakeholders’ perceptions and attitudes towards—SRMNCAH issues in Bangladesh, SRMNCAH service provision in Bangladesh for refugee populations, current reporting practices on SRMNCAH indicators, feasibility of reporting on the candidate set of core SRMNCAH indicators; and also, the necessary buy-in needed from the sector to successfully nationally scale up, endorse and report against these indicators.^3^

### Desk review

The project was initiated with a comprehensive review of peer-reviewed literature, existing published and unpublished data, including institutional and donor reports that focused on SRMNCAH indicators’ reporting and analysis in Bangladesh; coupled with an in-depth examination of the National SRMNCAH indicators’ list that organizations are required to report against. This desk review also helped informed the selection of the target populations for each of the KIIs and FGDs.

### Field-level assessment

#### Key informant interviews (KIIs)

We compiled a list of key agencies working and providing SRMNCAH services to FDMNs and the positions/key personnel responsible for data collection/ management in those agencies in Cox’s Bazar, Bangladesh. We included a number of national and international non-governmental organizations, government entities, and community-based organizations in the list to better understand the distribution of resources and feasibility across different entities. We purposively selected representatives from the staff of the listed agencies who were involved in either data management and processing or using data for decision making. We also consulted with WHO Dhaka and Cox’s Bazar Offices and UNFPA Cox’s Bazar office (leads SRHR sub-sector) for selecting and inviting organizational representatives for key informant interviews (KIIs). We conducted KIIs until achieving data saturation level. Thus, NF, AK, AA, BA, KM and TH led the data collection processes in the field and conducted key informant interviews with 24 representatives from 18 different entities between January–February 2020 using a semi-structured interview guide. As the majority of the agencies’ local headquarters were based in Cox’s Bazar, most of the interviews took place individually or in small groups in Cox’s Bazar. Some KIIs were also conducted online based on the availability of the interviewees during data collection period. KIIs focused on KIs’ perceptions and attitudes towards: SRMNCAH issues in Bangladesh, SRMNCAH service provision in Bangladesh for FDMNs, current reporting practices on SRMNCAH indicators, and the feasibility of reporting on the candidate set of core SRHMNAH indicators. We also explored stakeholders’ perceptions and attitudes of current challenges in documenting and resources needed to successfully report against these indicators. We, further, explored the necessary buy-in needed from among donor, governmental, and non-governmental agencies to enable the success of this effort. Informed written consents were taken from all interviewees before each KI.

#### Facility assessments

We conducted eight facility assessments at three primary, four secondary, and one community outreach center that provide SRMNCAH services to FDMNs. All the facilities were either inside or close to the Rohingya camps in Ukhiya sub-district and were identified as the largest SRMNCAH service providers to FDMNs in Cox’s Bazar. Prior, to these assessments, WHO sought permission from the Civil Surgeon’s Office and the Refugee Relief and Repatriation Commissioner’s (RRRC) Office in Bangladesh and the authorities of the selected facilities, following which the local research partner, BRAC James P Grant School of Public Health, BRAC University contacted each selected facility prior to the assessment. The assessment aimed to determine the nature and extent of SRMNCAH services offered, the ways in which patient information was collected, logged, stored, and safeguarded, and the types of human and technological resources used in data capture. In conjunction with the facility assessments, observational sessions were also carried out in all eight service sites to assess existing resources currently being employed to collect data and additional resources needed to collect additional needed data for the core set of SRMNCAH indicators as well as ethical, and privacy practices associated with data collection and storage at select facilities.

#### Focus group discussions

Finally, we conducted two focus group discussions with 16 frontline community health workers and their supervisors from the selected primary and secondary health centers where facility assessment took place. Participants provided verbal consent at the beginning of each FGD, which lasted an average of 90 min and took place in local Rohingya language. With consent, we audio-recorded FGDs, debriefed as a team after each discussion, and wrote analytic memos to capture group dynamics and identified early themes. The FGDs aimed to gain an understanding of the frontline providers on the: SRMNCAH needs and status of service provision in Bangladesh for forcibly displaced populations, current reporting practices of existing SRMNCAH indicators, and the feasibility of reporting on the proposed candidate set of core. Verbal consent was provided prior to each FGD from the participants, each FGD lasted for 90 min, and it took place in the local Rohingya Arabic language.

### Analytic approach

We employed an iterative, multi-phased approach to analyze the data, such that analysis occurred simultaneously with data collection [20, 21]. All the interviews and FGDs were audio recorded and later transcribed verbatim in the language of conducting interviews. Bangla transcripts were then translated into English. We also formally memoed after each encounter, a process that allowed for ongoing identification of emerging themes and patterns. We used NVivo software to manage our data comprising transcripts, notes, and memos. We used both inductive and deductive approaches for coding data using NVivo software. A coding framework was developed based on the objectives of the study. Then new emerging codes/themes were included in the coding framework. A group of trained experienced researchers analyzed data including coding, intercoder reliability checking, identifying themes and patterns and triangulating data collected through multiple methods. For analyzing participants responses on the proposed list of indicators, we manually entered collected data by organizations against each indicator in a checklist is Microsoft Excel and did descriptive analysis using analysis functions of Microsoft Excel. All electronic database were password protected and also stored in password protected computers. Feedback from the WHO led to the final recommendations. The analysis focused on the four core elements: 1) Feasibility of collecting the proposed core set of SRMNCAH indicators, 2) Relevance and usefulness of SRMNCAH data management mechanisms; 3) available existing resources and systems for national and humanitarian SRMNCAH data collection; and 4) ethical considerations of collecting and storing data.

### Research ethics

The Research Project Review Panel (RP 2) of the WHO-Department of Sexual and Reproductive Health reviewed and approved this study. Additionally, we obtained authorization to conduct this study from the Institutional Review Board (IRB) of BRAC James P Grant School of Public Health, BRAC University (IRB Reference No. 2019–033-IR). The Social Sciences and Humanities Research Ethics Board of the University of Ottawa also provided ethical approval (Protocol number: S-08-18-1029).

## Findings

### Feasibility of collecting proposed core set of SRMNCAH indicators

The findings of this assessment indicated that 48% of the proposed indicators were considered relevant and feasible to collect); many of the sexually transmitted infections (STIs) and reproductive tract infections (RTIs) (100%), newborn (81%), contraception (75%), maternal (53%), and abortion (40%) indicators were perceived to be relevant and feasible (see Tables 1, 2). Among the proposed indicators, 45% were reportedly collected during this study's data collection period. About 14% of indicators that were not collected could potentially be collected with available resources and training (see Table 2). The findings also suggest that 48% of the proposed indicators were deemed unfeasible or irrelevant—the child (30%) and adolescent health (17%), all HIV, prevention from mother to child (PMTC) indicators would not be feasible to collect.

**Table 1:** Feasibility of the proposed core set of SRMNCAH indicators in Bangladesh context

	Number of indicators by domain (n)	Number of indicators relevant to the Bangladesh context (n)	% Of indicators that are feasible
Contraception	4	4	100%
Comprehensive Abortion Care	5	3	60%
Maternal Health	17	17	100%
Newborn Health	16	16	100%
Child Health	10	8	80%
Adolescent health	6	3	50%
Sexual and gender-based violence	7	4	57%
HIV	3	0	0%
Prevention from Mother to Child	4	1	25%
Sexually transmitted infections (STIs) and reproductive tract infections (RTIs)	1	1	100%
Total	73	57	78%

Necessary ﻿modifications*

**Table 2:** Summary findings of the feasibility of collecting the following proposed SRMNCAH indicators in the humanitarian context of Bangladesh

No.		Indicator name	Overall % of agencies reporting	Place of collection	Facilitators to routine collection	Barriers to routine collection		Resources needed for routine collection	Exclude/include
Contraception									
1.1		Number of clients initiating contraception	82%	Health facilities and organizations providing reproductive health services	Mandatory reporting to the health sector, MoHFW and the SRHWG; National reporting systems: DHIS2, EWARS	Lack of harmonized reporting system leads to increased strain, duplication of data and unreliable data	*# of clients receiving a contraceptive service, by method* Clarification on the wording surrounding "initiating" Standardize definition of new user vs. recurrent user	Resources for integration into HIS/existing data collection systems Enable and implement methods to track specific patients at the facility and community level	Include
1.2		Number of clients receiving emergency contraception	50%	Health facilities and organizations providing reproductive health services	Mandatory reporting to the health sector, MoHFW and the SRHWG; National reporting systems: DHIS2, EWARS	Socio-cultural barriers Low and unreliable availability of commodities: limited provision of EC for cases that require CMR	*# of clients receiving CMR services within 120 h*	Resources and training regarding security and data privacy Resources for integration into DHIS2 Resources needed to enable coordination efforts for the SRHWG	Include
1.3		Percentage of clients adopting modern contraceptive method after delivery	0%	Secondary and tertiary facilities only	Mandatory reporting to the health sector, MoHFW and the SRHWG; National reporting systems: DHIS2, EWARS	Unreliable population metrics since reporting systems cannot track individual service users Applicable in secondary and tertiary facilities only	*# of clients adopting a modern contraceptive method after delivery* Removal of denominator	N/A	Include
1.4		Percentage of clients adopting modern contraceptive method after abortion	0%	N/A	N/A	Socio-cultural barriers Legal status of abortion in Bangladesh	*# of clients adopting a modern contraceptive method after menstrual regulation* Change "abortion" to "menstrual regulation" Couple indicator with an indicator tracking the number of outreach activities from the CHWs surrounding menstrual regulation Removal of denominator	Resources and training regarding security and data privacy Encryption and coding of sensitive information is needed Enabling policies to ensure confidentiality Training for CHW on security and data privacy measures in place and communicating these to the Rohingya community	Include
Comprehensive abortion care									
2.1	Number of clients requesting an abortion		0%	N/A	N/A	Potential risk for client and primary care provider Legal status of abortion in Bangladesh Socio-cultural barriers Insufficient data security and privacy	N/A	N/A	Exclude
2.2	Number of clients receiving an abortion referral		0%	N/A	N/A	Gaps in coordination between health service providers and referrals Potential risk for patient and primary care provider Legal status of abortion in Bangladesh Socio-cultural barriers Insufficient data security and privacy	N/A	N/A	Exclude
2.3	Number of clients receiving an induced abortion		27%	N/A	N/A	Socio-cultural barriers cause limited service-provision, underreporting and hesitancy in the Rohingya community Insufficient data security and privacy measures set in place Potential risk for client and primary care provider	*# of clients receiving menstrual regulation services, disaggregated by age (10–13;13–15;15–18; and equal or greater than 18)* Change "abortion" to "menstrual regulation"	Training on data security and data privacy Encryption and coding of sensitive information is needed Enabling policies to ensure confidentiality Training for CHW on security and data privacy measures in place and communicating these to the Rohingya community	Include
2.4	Number of clients presenting for post-abortion care (PAC)		55%	Secondary and tertiary facilities only	National and humanitarian reporting systems: DHIS2, EWARS, SRHWG, KOBO	Applicable in secondary and tertiary facilities only Socio-cultural barriers Insufficient data security and privacy Potential risk for patient and primary care provider	N/A	N/A	Include
2.5	Number of clients receiving PAC		64%	Secondary and tertiary facilities only	National and humanitarian reporting systems: DHIS2, EWARS, SRHWG, KOBO	Applicable in secondary and tertiary facilities only Socio-cultural barriers Insufficient data security and privacy Potential risk for patient and primary care provider	N/A	N/A	Include
Maternal health									
3.1	Number of maternal deaths		82%	Secondary and tertiary facilities	Organizations with allocated funding for data collection developed comprehensive HIS for accurate maternal data collection National and humanitarian reporting systems: DHIS2, EWARS, SRHWG, KOBO	Lack of centralized system to collect quality and timely maternal death data at the facility and community levels	*# of maternal deaths in the facility, by cause of death* Disaggregate indicator by cause Couple indicator with indicators that capture maternal death in the community	Increased transparency in auditing practices Resources needed to develop and implement at the community level to capture the indicators for the maternal deaths in the community	Include
3.2	Number of maternal deaths, disaggregated		73%	Secondary and tertiary facilities	Organizations with allocated funding for data collection developed comprehensive HIS for accurate maternal data collection National and humanitarian reporting systems: DHIS2, EWARS, SRHWG, KOBO	Lack of centralized system to collect quality and timely maternal death data at the facility and community levels	*# of maternal deaths in the facility, disaggregated by age (10–13;13–15;15–18; and equal or greater than 18)*	Training for data collectors on the different causes of maternal death and how to encode for each Develop detailed manuals for frontline workers and data collection Training and capacity building for community health workers Increased transparency in auditing practices surrounding maternal deaths	Include
3.3	Percentage of maternal death reviews		73%	Secondary and tertiary facilities	Routinely collected by secondary and tertiary facilities Organizations with allocated funding for data collection developed comprehensive HIS for accurate maternal data collection National and humanitarian reporting systems: DHIS2, EWARS, SRHWG, KOBO	Lack of centralized system to collect quality and timely maternal death data at the facility and community levels	*# of maternal deaths in the facility that were audited and reviewed* Disaggregate indicator by cause Couple indicator with indicators that capture maternal death in the community Removal of denominator	Training of data collectors on the different causes of maternal death and how to encode for each Develop detailed manuals for frontline workers and data collectors Training and capacity building for community health workers Increased transparency in auditing practices surrounding maternal deaths Enable and implement methods and policies to ensure coordination between agencies and systematic undertaking of maternal death reviews	Include
3.4	Number of clients receiving antenatal care (ANC)		64%	Secondary and tertiary facilities	Routinely collected by secondary and tertiary facilities		N/A	N/A	Include
3.5	Number of deliveries		73%	Secondary and tertiary facilities	Routinely collected by secondary and tertiary facilities Community health workers promote community members to opt for facility-based deliveries	No birth registries at the facility level Gaps in policies and systems: lack of national civil registry policies for refugees	*# of clients delivering in a facility, including both live and stillbirths* Couple indicator with indicators to capture births occurring in the community	Training for frontline workers on stillbirths Leveraging systems to capture stillbirths for community births (UNICEF) Training and capacity building for community health workers	Include
3.6	Number of deliveries, disaggregated		55%	Secondary and tertiary facilities	Routinely collected by secondary and tertiary facilities Community health workers promote community members to opt for facility-based deliveries	No birth registries at the facility level Gaps in policies and systems: lack of national civil registry policies for refugees	*# of clients delivering in facility, including both live and stillbirths, disaggregated by age (10–13;13–15;15–18; and equal or greater than 18)*	Training for frontline workers on stillbirths Leveraging systems to capture stillbirths for community births (UNICEF) Training and capacity building for community health workers	Include
3.7	Number of clients receiving post-natal care (PNC)		73%	Secondary and tertiary facilities	Routinely collected by secondary and tertiary facilities Community health workers promote community members to opt for facility-based deliveries		*# of clients receiving post-natal care, disaggregated between 2 and 7 days*	N/A	Include
3.8	Number of caesarean section deliveries		64%	Secondary and tertiary facilities	Routinely collected by secondary and tertiary facilities	Lack of training/equipment to provide service in some facilities Applicable in secondary and tertiary facilities only	# of caesarian section deliveries, disaggregated by medically or nonmedically necessary Couple indicator with an indicator on the number of referrals for caesareans	Resources needed to improve monitoring referral systems within the camp	Include
3.9	Availability of PAC		0%	N/A	N/A	Lack of training/equipment to provide PAC in some facilities Socio-cultural barriers Insufficient data security and privacy Potential risk for client and primary care provider	N/A	Service mapping of PAC providers Provide specific contours on when, how and by whom these should be collected	Include
3.10	Availability of basic emergency obstetric care (BEmOC)		0%	N/A	N/A		N/A	Service mapping of facilities Provide specific contours on when, how and by whom these should be collected	Include
3.11	Availability of comprehensive emergency obstetric care (CEmOC)		0%	N/A	N/A	Lack of training/equipment to provide CEmOC in health centres Applicable in secondary and tertiary facilities only	N/A	Service mapping of facilities Provide specific contours on when, how and by whom these should be collected	Include
3.12	Availability of skilled personnel		0%	N/A	N/A		N/A	Service mapping of facilities	Include
3.13	Number of antenatal care clients with tetanus vaccination		55%	Administered by health sector/state	Administered by the health sector/state	Vaccination is typically administered by the state	N/A	Buy-in among immunization teams and immunization reporting systems Resources needed to integrate the health system into data collection systems	Include
3.14	Number of ANC clients receiving preventive therapy for malaria		27%	Administered by certain NGOs	Administered by certain NGOs	Service not routinely provided Clients are referred to facilities that offer malaria therapy, but data is not usually collected	N/A	N/A	Include
3.15	Number of ANC clients receiving syphilis screening		36%	Secondary and tertiary facilities	National and humanitarian reporting systems: DHIS2, EWARS, SRHWG, KOBO Can be collected under cervical cancer screening	Lack of training/equipment to provide service: Not all facilities are equipped with lab equipment and materials for screening practices	Clarification on the term "screening". There is confusion on whether screening includes a test or not	Develop detailed manuals (with specific definitions) for frontline workers and data collectors	Include
3.16	Number of ANC clients receiving urinary tract infection screening or treatment		45%	Secondary and tertiary facilities	National and humanitarian reporting systems: DHIS2, EWARS, SRHWG, KOBO	Lack of training/equipment to provide service: Not all facilities are equipped with lab equipment and materials for screening practices	Clarification on the term "screening". There is confusion on whether screening includes a test or not	Develop detailed manuals (with specific definitions) for frontline workers and data collectors	Include
3.17	Number of clients with identified maternal morbidities during post-natal care (PNC)		27%		National and humanitarian reporting systems: DHIS2, EWARS, SRHWG, KOBO		*# of clients identified maternal morbidities, by type of morbidity, during post-natal care*	Develop detailed manuals for frontline workers and data collectors on the different types of agreed upon morbidities (outline definitions for accurate reporting)	Include
Newborn health									
4.1	Number of neonatal deaths		73%		National and humanitarian reporting systems: DHIS2, EWARS, SRHWG, KOBO	No birth registries at the facility level: Facilities do not have access to number of babies registered Gaps in policies and systems: Lack of national civil registry policies for refugees	*#of neonatal deaths (0–28) at the facility level* Couple indicator with an indicator tracking neonatal death within the community Couple indicator with the age of the mother given the high rates of early marriage	Training for community health workers on recording neonatal deaths within the community	Include
4.2	Number of stillbirths		55%		National and humanitarian reporting systems: DHIS2, EWARS, SRHWG, KOBO	No birth registries at the facility level: Facilities do not have access to number of babies registered Gaps in policies and systems: Lack of national civil registry policies for refugees	Couple indicator with the age of the mother given the high rates of early marriage	Training for community health workers on recording neonatal deaths within the community	Include
4.3	Number of babies born low birth weight		73%		National and humanitarian reporting systems: DHIS2, EWARS, SRHWG, KOBO	No birth registries at the facility level: Facilities do not have access to number of babies registered Gaps in policies and systems: Lack of national civil registry policies for refugees	*# of babies born low birth weight, disaggregated by age of mother* Couple indicator with the age of the mother given the high rates of early marriage Couple indicator tracking malnutrition among pregnant women	Training for community health workers on recording neonatal deaths within the community	Include
4.4	Number of small and sick newborns receiving care		55%		National and humanitarian reporting systems: DHIS2, EWARS, SRHWG, KOBO	N/A	Couple indicator with the age of the mother given the high rates of early marriage	N/A	Include
4.5	Number of newborns receiving post-natal care		55%		National and humanitarian reporting systems: DHIS2, EWARS, SRHWG, KOBO	N/A	N/A	N/A	Include
4.6	Availability of KMC		18%		KMC is a priority program due to premature and low birth weight deaths	N/A	N/A	Service mapping of facilities	Include
4.7	Availability of neonatal resuscitation		55%	Secondary and tertiary facilities only	Captured and administered at the secondary and tertiary facilities	Applicable in secondary and tertiary facilities only	N/A	Service mapping of facilities	Include
4.8	Number of neonatal deaths, disaggregated		45%		National and humanitarian reporting systems: DHIS2, EWARS, SRHWG, KOBO	Indicator is collected but not disaggregated	N/A	Training for data collectors on the different causes of neonatal death and how to encode for each Develop detailed manuals for frontline workers and data collectors Training and capacity building for community health workers Increased transparency in auditing practices surrounding maternal deaths Enable and implement methods and policies for coordination and systematic collection of neonatal deaths	Include
4.9	Percentage of perinatal death reviews		36%		Cause of death is recorded	No formal audit of collected data	N/A	Training for primary care providers on capturing and recording perinatal death and reviews for cause of death Training for community health workers on the system for reporting deaths occurring within the community	Include
4.1	Number of newborns receiving Hepatitis B vaccine		18%		N/A	Service not routinely provided: Hepatitis B doses are not part of all immunization schedules in Bangladesh	N/A	Buy-in among immunization teams and integration of immunization data collection systems at the facility and community level	Include
4.11	Number of newborns initiating breastfeeding early		27%		National and humanitarian reporting systems: DHIS2, EWARS, SRHWG, KOBO	N/A	*# of newborns initiating breastfeeding before discharge*	N/A	Include
4.12	Number of infants weighed at birth		36%		National and humanitarian reporting systems: DHIS2, EWARS, SRHWG, KOBO	N/A	N/A	Resources needed to establish equipment to weigh babies in all facilities and appropriate equipment for community health workers	Include
4.13	Number of babies registered		0%	N/A	N/A	Gaps in policies and systems No birth registries at the facility level: Facilities do not have access to the number of babies registered	N/A	N/A	Exclude
4.14	Number of newborns receiving treatment for possible severe bacterial infection (PSBI)		27%	Secondary and tertiary facilities only	Captured and administered at the secondary and tertiary facilities	Applicable in secondary and tertiary facilities only	N/A	N/A	Include
4.15	Number of newborns admitted		27%			Service not routinely provided: Only certain facilities have a NICU or KMC unit	Couple indicator with number of referrals	N/A	Include
4.16	Number of newborns with morbidities identified during PNC		27%				N/A	Resources for primary care providers on the definitions for morbidity type Extensive training and capacity building will need to be implemented	Include
Child health									
5.1	Number of deaths of children under 5		45%	Health facilities, primary, and secondary health services	National and humanitarian reporting systems: DHIS2, EWARS, SRHWG, KOBO	Unreliable population metrics since reporting systems cannot track individual service users and deaths outside of the facility	Removal of denominator	Training of CHW and system for reporting deaths occurring within the community Leveraging systems to capture the child health indicators within the community (UNICEF)	Include
5.2	Under 5 mortality rate		0%	N/A	N/A	Population-level indicator with impractical denominator Unreliable population metrics since reporting systems cannot track individual service users and deaths outside of the facility	N/A	N/A	Exclude
5.3	Percentage of children under 5 with suspected pneumonia taken to appropriate health facility		36%	Health facilities, primary, and secondary health services	National and humanitarian reporting systems: DHIS2, EWARS, SRHWG, KOBO	N/A	Standardize definition of acute respiratory infection (ARI) Clarification on the term “survey”	Develop detailed manuals for frontline workers and data collectors on indicator definition Leveraging systems to capture the child health indicators within the community (UNICEF)	Include
5.4	Coverage of diarrhea treatment		55%		National and humanitarian reporting systems: DHIS2, EWARS, SRHWG, KOBO	N/A	N/A	Leveraging systems to capture the child health indicators within the community (UNICEF)	Include
5.5	Percentage of children under 5 who are wasted		36%		National and humanitarian reporting systems: DHIS2, EWARS, SRHWG, KOBO	Population-level indicator with impractical denominator	*# of children under 5 who are wasted* Removal of denominator		Include
5.6	Percentage of children under 5 who are registered		0%	N/A	N/A	Gaps in policies and systems No registration of individuals at the facility level: Registration of individuals occurs through the state; facilities do not have access to this information	Removal of denominator	N/A	Exclude
5.7	Number of children presenting with fever tested for malaria in endemic settings		45%	Administered by health sector/state	The health sector performs active surveillance of malaria cases in the region	N/A	N/A	N/A	Include
5.8	Number of confirmed cases of malaria in endemic settings		55%	Administered by health sector/state	The health sector performs active surveillance of malaria cases in the region	N/A	N/A	N/A	Include
5.9	Percentage of confirmed malaria cases treated		36%	Administered by health sector/state	The health sector performs active surveillance of malaria cases in the region	N/A	*# of confirmed malaria cases treated* Removal of denominator	Enable and implement methods to track specific patients at facility and community level	Include
5.1	Coverage of DPT3		36%	Administered by health sector/state	Captured and administered at the state level	Population-level indicator with impractical denominator	Removal of denominator	Buy-in among immunization teams and systems	Include
Adolescent health									
6.1	Adolescent birth rate		64%	N/A	Data can be extracted from DHIS2 and SRHWG reports	Gaps in policies and systems: No current data collection mechanism in place for adolescent health indicator reporting Stakeholders do not collect the exact age of the patient Socio-cultural barriers	*# of adolescents giving birth, disaggregated by age (10–13;13–15;15–18)* Removal of denominator	Resources needed to integrate adolescent health indicators into routine service delivery as a specific area of its own, including for data collection to ensure reliability and validity of the data Resources needed to invest in the adolescent task force to enable camp wide coordination of data capturing and analysis	Include
6.2	Sexual violence against children		0%	N/A	N/A	Information not actionable Gaps in policies and systems: No current mechanisms of data collection for adolescent health indicator reporting Socio-cultural barriers	N/A	N/A	Exclude
6.3	Adolescent mortality rate		36%	N/A	Data can be extracted from DHIS2 and SRHWG reports	Gaps in policies and systems: No current mechanisms of data collection for adolescent health indicator reporting Indicator collected but not all stakeholders disaggregate data	*# of adolescent death, disaggregated by age (10–13; 13–15;15–18)* Removal of denominator	Resources needed to integrate adolescent health indicators into routine service delivery as a specific area of its own, including for data collection to ensure reliability and validity of the data Resources needed to invest in the adolescent task force to enable camp wide coordination of data capturing and analysis	Include
6.4	Percentage of adolescents living with HIV who are currently receiving antiretroviral therapy, disaggregated		0%	N/A	N/A	Specific infectious disease reporting requirements and management protocols for individual cases of HIV Population-level indicator with impractical denominator Unreliable population metrics since reporting systems cannot track individual service users Gaps in policies and systems: No current mechanisms of data collection for adolescent health indicator reporting	N/A	N/A	Exclude
6.5	Immunization coverage rate		36%	Administered by health sector/state	Data can be extracted from DHIS2 and SRHWG reports Not all facilities collect this information, but it is accessible at the health sector/state level	Gaps in policies and systems: No current mechanisms of data collection for adolescent health indicator reporting Vaccination generally administered at the state level	*# of adolescents receiving the nationally mandated immunization* Removal of denominator	Resources needed to integrate adolescent health indicators into routine service delivery as a specific area of its own, including for data collection to ensure reliability and validity of the data Resources needed to invest in the adolescent task force to enable camp wide coordination of data capturing and analysis	Include
6.6	Suicide rate, disaggregated		0%	N/A	N/A	Information not actionable Population-level indicator with impractical denominator Unreliable population metrics since reporting systems cannot track individual patients Gaps in policies and systems: No current mechanisms of data collection for adolescent health indicator reporting Socio-cultural barriers	N/A	N/A	Exclude
Sexual and gender-based violence									
7.1	Number of rape survivors		36%		National and humanitarian reporting systems: DHIS2, EWARS, SRHWG, KOBO	Socio-cultural barriers cause gaps in reporting	*# of clients receiving CMR services, disaggregated by sex and age* Remove term "rape survivors"	Training on data security and & data privacy Encryption and coding of sensitive information is needed Enabling policies to ensure confidentiality Training of CHW of security and data privacy measures in place and communicating these to the Rohingya community	Include
7.2	Percentage of health facilities with clinical management of rape services		0%	N/A	Data could be extracted through data from patient files	Lack of training/equipment to provide service: Insufficient community outreach mechanisms for SGBV service availability leads to under-reporting/underutilization of services Socio-cultural barriers	N/A	Service mapping of facilities	Include
7.3	Percentage of rape survivors receiving HIV post-exposure prophylaxis		0%	Administered by health sector/state	Collected at the state level	HIV surveillance and treatment is overseen by the state	N/A	N/A	Exclude
7.4	Percentage of rape survivors receiving emergency contraception		36%		National and humanitarian reporting systems: DHIS2, EWARS, SRHWG, KOBO	Indicator is collected but data is not disaggregated by method	*# of clients receiving CMR services disaggregated by method, sex and age* Removal of denominator	Training on data security and & data privacy Encryption and coding of sensitive information is needed Enabling policies to ensure confidentiality Training for CHW on security and data privacy measures in place and communicating these to the Rohingya community	Include
7.5	Number of rape survivors requesting abortion		0%	N/A	N/A	Potential risk for the client Insufficient security and privacy measures set in place Socio-cultural barriers Legal status of abortion in Bangladesh Service not routinely provided	N/A	N/A	Exclude
7.6	Number of rape survivors receiving induced abortion care or referral		0%	N/A	N/A	Gaps in coordination between service providers and referrals Potential risk for patient and primary care provider Insufficient data security and privacy Socio-cultural barriers Legal status of abortion in Bangladesh	N/A	N/A	Exclude
7.7	Availability of intimate partner violence front line support (LIVES)		36%	Health facilities	Part of the community outreach agenda	Lack of infrastructure and absence of private spaces is not conducive to confidentiality and safety for women and girls	N/A	Service mapping of facilities Training on data security and data privacy Encryption and coding of sensitive information is needed Enabling policies to ensure confidentiality Training for CHW on security and data privacy measures in place and communicating these to the Rohingya community	Include
HIV									
8.1		Antiretroviral therapy coverage among people living with HIV, disaggregated	0%	Administered by health sector/state	Captured and administered at the state level	Specific infectious reporting requirements and management protocols for individual cases: Strict anonymity and coding of HIV cases Sociocultural barriers	N/A	N/A	Exclude
8.2		Percentage of exposed individuals receiving post-exposure prophylaxis	0%	Administered by health sector/state	Captured and administered at the state level	Specific infectious reporting requirements and management protocols for individual cases: Strict anonymity and coding of IV cases Sociocultural barriers	N/A	N/A	Exclude
8.3		Percentage of donated blood units screened for HIV in quality assured manner	0%	Administered by health sector/state	Captured and administered at the state level	Specific infectious reporting requirements and management protocols for individual cases: Strict anonymity and coding of HIV cases Sociocultural barriers Applicable in secondary and tertiary facilities only Lack of training/equipment to provide service: Insufficient screening tools	N/A	N/A	Exclude
Prevention of mother-to-child transmission									
9.1		Percentage of antenatal care clients receiving syphilis screening and treatment	36%	Administered by certain NGOs and health facilities	Syphillis screening can also occur during cervical cancer screening	Lack of training/equipment to provide service (at all facilities): Insufficient lab equipment and materials for screening procedures	Clarification on the term "screening". There is confusion on whether screening includes a test or not Removal of denominator	Training on data security and data privacy Encryption and coding of sensitive information is needed Enabling policies to ensure confidentiality Training for CHW on security and data privacy measures in place and communicating these to the Rohingya community	Include
9.2		Percentage of antenatal care clients offered testing for HIV	0%	Administered by health sector/state	N/A	Specific infectious disease reporting requirements and management protocols for individual cases	N/A	N/A	Exclude
9.3		Percentage of HIV-positive pregnant people receiving antiretroviral therapy	0%	Administered by health sector/state	N/A	Specific infectious disease reporting requirements and management protocols for individual cases	N/A	N/A	Exclude
9.4		Percentage of all deliveries to HIV-positive mothers receiving antiretrovirals	0%	Administered by health sector/state	N/A	Specific infectious disease reporting requirements and management protocols for individual cases	N/A	N/A	Exclude
Sexually transmitted infections (STIs) and reproductive tract infections (RTIs)									
10.1		Percentage of STI/RTI cases managed	82%		National and humanitarian reporting systems: DHIS2, EWARS, SRHWG, KOBO	Socio-cultural barriers prevent adequate treatment of STI cases	*# of patients with STI/RTI accessing services who are diagnosed symptomatically, and counselled according to protocol* Clarification between the number of cases and the number of cases “managed” STI and RTI cases need to be formulated as separate indicators Removal of the denominator	Training on data security and data privacy Encryption and coding of sensitive information is needed Enabling policies to ensure confidentiality Training for CHWs on security and data privacy measures in place and communicating these to the Rohingya community	Include

Texts in italic indicates new indicators recommended to add in the core set of indicators for Bangladesh context

The study found several harmonized national and international reporting systems in place to capture information for certain SRMNCAH components, yet the resources and systems for data collection are fragmented and inconsistent. Access to comprehensive and user-friendly computerized reporting systems, adequate and trained staff, and available resources, materials/tools and internal capacity varied between agencies and those fragmented reporting processes and insufficient human resources have proliferated the duplication of information. The findings also indicate gaps in resources and systems, including internal capacity, funding, and materials. Irrespective of access to resources, challenges with infrastructure at the ground level impedes the quality of upstream data distribution and analysis. Although, several organizations have built dedicated internal health information systems, access to funding and/or human and technological capacities varied between agencies. Due to the scarcity of resources, the abundance of reporting systems, and a lack of national buy-in, organizations in camp-based settings had to input and analyze their data manually, which ultimately negatively impacted data quality.

While stakeholders expressed the need for an overall harmonized list of SRMNCAH indicators, some of the proposed indicators in the framework had varying levels of feasibility in the local context. Overall, stakeholders expressed concern about the length of the list and data for the challenges surrounding the collection of indicators containing a population-level denominator given the absence of systems to track individual patients. Table 2 provides an overview of the included and excluded list of indicators, the reported percentage of agencies currently collecting data on the indicators; the site of data collection; their respective facilitators and barriers for routine data collection; any necessary modifications and resources needed for routine data collection.

The results indicate that some contraceptive methods related indicators (indicators 1.1–4 in Table 2) could be feasible for inclusion in some conditions—availability of resources for integration into existing data collection systems, an electronic tracking system in place to keep track of patients’ use at the facility and community level, contextual rephrasing/reframing of the indicators and training on data privacy. The legal status of abortion in Bangladesh makes routine collection of abortion data difficult as it creates a potential risk for patients and providers alike, yet stakeholders reported the possibility for inclusion if the indicator is reframed from abortion to menstrual regulation (MR)^4^for all indicators except for indicators 2.1–2 in Table 2.

“…*At this point, abortion is not legal. But the approved term here is menstrual regulation. So, we are providing menstrual regulation and post-abortion care, and on site, we are emphasizing very much on family planning*”—explained an iNGO representative. There was no centralized system to report maternal death and no civil registry policies for refugees. In a FGD with an iNGO community health workers and their supervisors, participant 1 noted, “*Registration is a problem here. For host communities, registration is done. There is a civil registration system for our general people but for forcibly displaced Myanmar nationals, the camp administration manages it*”. However, stakeholders reported that all maternal health indicators (3.1–3.17 in Table 2) could be included with additional resources and training. Most of the newborn health indicators (4.1–4.8 in Table 2) rely on facility-based information and therefore are either currently collected or could easily be collected. However, stakeholders expressed concerns about the way neonatal deaths (indicator 4.1) and stillbirth (indicator 4.2) are defined, recorded, and audited, particularly at the community level. A national NGO representative explained “…*we don’t have birth registries here [at the facility level]*.” Most of the child health indicators (5.1, 5.3–5.5, 5.7–5.10, Table 2) are currently being collected or could easily be collected with the removal of the denominator due to the absence of population-level data. Adolescent health indicators are in the development phase but could be extracted from the DHIS2 if age is disaggregated to reflect the rates of early marriage among Rohingya refugee girls. Our stakeholders believed that indicators 6.2, 6.4 and 6.6 on adolescent health (Table 2) would not be feasible to collect due to cultural barriers and should be removed.

There are significant gaps in data regarding the sexual and gender-based violence (SGBV) related indicators in this camp-based setting. Indicators 7.3, 7.5 and 7.6 (Table 2) were recommended for exclusion due to potential risks to the patient and socio-cultural barriers. Stakeholders raised concerns about including the HIV/AIDS-related indicators (8.1 and 8.2 in Table 2) due to the significant stigma and discrimination from service providers, and concurrently, all HIV-AIDS cases are referred to the district hospital under the Ministry of Health (MoH). “…*HIV program, they [the government] keep this data…And they have some centers, they provide this and medications*. *There is no clear guidance from the government right now for how to handle these cases in the camps*.”—explained a representative from an iNGO (KII Participant 1). Similarly, to the findings for the HIV domain, government regulations prevent service delivery and therefore impede data collection efforts on the PMTCT indicators. (9.2–9.4). STIs and RTIs are not being consistently differentiated through their current indicator reporting and as such language for indicator 10.1 could be clarified. Table 2 presents additional information about the indicators proposed for each topic and a detailed narrative in WHO’s Bangladesh country-level report.

### Relevance and usefulness of humanitarian SRMNCAH data management mechanisms

#### Perceived advantages with current and proposed SRMNCAH indicator reporting

All of the participants agreed that accurate and reliable SRMNCAH data provide the opportunity to implement evidence-based programming, define priorities, identify emerging diseases, and ensure accountability among implementers, which ultimately could lead to improved health outcomes of FDMNs in this context. Other KIs noted that the collection and reporting of SRMNCAH indicators allow organizations to identify programs and interventions that were successful in increasing the FDMNs healthcare seeking behaviors. As indicated by a cluster representative (KII participant 2), “*In general, in the last few years, we have seen an increase in FDMNs coming to the facilities. Many of the partners have worked hard to increase facility births and bringing women to the clinics. So, we want to see what programs and projects worked.*” KIs also indicated that the collection and reporting of SRMNCAH indicators provides organizations the opportunity to monitor and evaluate the progression of facility-based service utilization, enabling patient triage as patients enter their facilities, and meet national and donor funding requirements.

There was strong consensus among the participants that there is a need for a harmonized list of SRMNCAH indicators to mitigate the challenges faced with the current fragmented monitoring and evaluation system. There was overall support for the general contours of the proposed list of indicators, and participants believed many of the indicators were aligned with national and internal data collection practices and priorities. The KIs also believed that some indicators that weren’t currently being collected could be incorporated into their current systems. Yet, they highlighted that the ease of including an indicator did not necessarily equate to the feasibility of reporting against the indicator. Overall, our participants felt that if we garnered enough support from donors, UN agencies, local government sectors, and international NGOs on this standardized list, it would serve as a valuable tool.

#### Perceived disadvantages with current SRMNCAH indicator reporting

Our findings also indicated some disadvantages in capturing certain SRMNCAH indicators, specifically those surrounding SGBV and comprehensive abortion care (CAC. Socio-cultural barriers and multi-level access issues are preventing the accurate information reporting required to justify the implementation of SGBV programming to meet the needs of the FDMN in this context. This is mainly attributed to the fact that very few cases are reported that falsely convey the actual burden of this problem. A gender-based violence (GBV) specialist (KII participant 3) explained,

“*There is a lot of under-reporting and delayed reporting for sexual violence for various reasons. We are also trying to address why people are reporting late for health services. But we do not want to force the community before we strengthen our health services… Because there are other barriers that are linked to the services themselves, like the quality of services. When people don’t have confidence in the services, or they don’t trust the provider, they may not report.*”

Another disadvantage was the lack of a rigorous tracking system for FDMNs; therefore, identifying patients and tracking service utilization, and outcomes are challenging. As described by a health care provider (KII participant 4) “*The challenge in the camp is that we do not have any way to identify a unique patient…we still do not have any health card as of now, like a health card by which we can say that this woman has received this number of services, she’s come this time – you know? Tracking that patient overall isn’t possible*.”

#### Perceived gaps in the proposed SRMNCAH indicators

##### Indicators that should be removed from the core set of SRMNCAH indicators

The study participants identified a number of proposed indicators that were not relevant or useful in the humanitarian context in Bangladesh and should therefore be removed. The rationale for their exclusion, as per the KIs, revolved around one or more of these reasons: (1) barriers in patient tracking; (2) national enforced regulations and protocols that restrict collecting information on certain issues, such as HIV/AIDS subject; (3) concerns surrounding patient privacy, confidentiality, and safety; and/or (4) impractical or unactionable applications. In addition, stakeholders were uniform in their belief that population-level indicators in Cox’s Bazar are completely unfeasible to collect; and require significant resources, funding, policy reforms. Investment in infrastructure and national registries would be required to capture a reliable population-level denominator and ensure FDMN privacy concerns are met. In Table 2 presents the list of the indicators that participants in the study identified for removal with their rationale for exclusion.

All stakeholders identified indicators associated with ‘referrals’ as not feasible due to gaps in coordination, infrastructure and a centralized referral capturing system and should therefore be removed. In addition to disparities in resources and systems to collect referral indicators, findings also suggest that issues related to patient tracking hinder the feasibility of collecting referral-related indicators. Other stakeholders indicated privacy and safety factors as important challenges that impede the collection of abortion and SGBV related indicators. A number of the FGD participants informed that the current infrastructure within the camp doesn’t provide a conducive environment for safe and confidential counselling for Rohingya women and girls. Key informants echoed the same infrastructural concerns and added that due to both low levels in GBV reporting and the accessibility of data on the HIS tool, which could lead to compromising patient privacy and confidentiality.

##### Additional indicators that should be added to the core set of SRMNCAH indicators

**Fig. 2:**
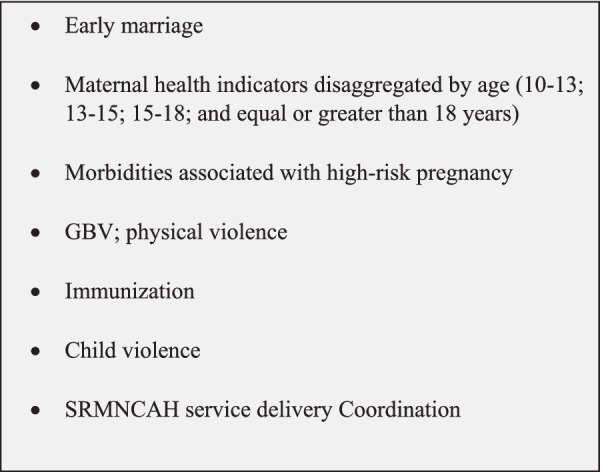
Additional topics proposed for inclusion in the core list of SRMNCAH indicators

Stakeholders in Bangladesh proposed some additional indicators for inclusion to the core SRMNCAH list. Those suggested indicators were mostly focused on GBV, adolescent pregnancy, immunizations and engagement of community health workers in SMNCAH response. For instance, due to the high prevalence of early marriage among the Rohingya community, participants believed that there should be cross-cutting indicators related to early marriage to better understand health outcomes associated with early marriage, particularly with early-pregnancy. They also highlighted that there should be further disaggregation of these indicators by age. In Fig. 2, we provide a list of additional topics that the stakeholders in Bangladesh perceived should be included on the core SRMNCAH indicator list.

### Existing systems and resources for collecting SRMNCAH indicators

The findings from our study indicate a great variety in available data collection information systems that international and national partner organizations develop, implement, and use to collect data on utilization of health services by FDMNs in Cox’s Bazar. A list of existing data collection resources and systems reported by our stakeholders is presented in Table 3. Access to comprehensive, user-friendly computerized systems, adequate staff, funding and capacity varied across agencies. The findings also show a number of harmonized national and international reporting systems that are used to capture information for certain SRMNCAH indicators, including, harmonized health information system (HIS), the District Health Information Software 2 (DHIS2) and working group tools, including the sexual and reproductive health (SRH), gender-based violence (GBV), and community health workers (CHW) working group tools. Our findings indicate that these fragmented reporting processes and insufficient human resources have proliferated the duplication of information.

**Table 3:** Existing monitoring and evaluations systems reported by our stakeholders, by domain of indicators

SRMNCAH domain	Existing system
Contraception	Patient charts and registries Tally sheets Internal HIS Community health workers (CHWs) Mobile app (tablets) DHIS2 EWARS SRHWG (Excel) KOBO
Comprehensive abortion care	Patient charts and registries Tally sheets Internal HIS Community health workers (CHWs) Mobile app (tablets) DHIS2 EWARS SRHWG (Excel) KOBO
Maternal health	Patient charts and registries Tally sheets Internal HIS Community health workers (CHWs) Mobile app (tablets) DHIS2 EWARS SRHWG (Excel) KOBO
Newborn health	Patient charts and registries Tally sheets Internal HIS Community health workers (CHWs) Mobile app (tablets) DHIS2 EWARS SRHWG (Excel) KOBO
Child health	Patient charts and registries Tally sheets Internal HIS Community health workers (CHWs) Mobile app (tablets) DHIS2 EWARS SRHWG (Excel) KOBO
Adolescent health	N/A
Sexual and gender-based violence (SGBV)	Patient charts and registries Tally sheets Internal HIS Community health workers (CHWs) Mobile app (tablets) DHIS2 EWARS SRHWG (Excel) KOBO
HIV	System controlled by the MOH
PMTCT	System controlled by the MOH
Sexually transmitted infections (STIs) and reproductive tract infections (RTIs)	Patient charts and registries Tally sheets Internal HIS Community health workers (CHWs) Mobile app (tablets) DHIS2 EWARS SRHWG (Excel) KOBO

**Fig. 3:**
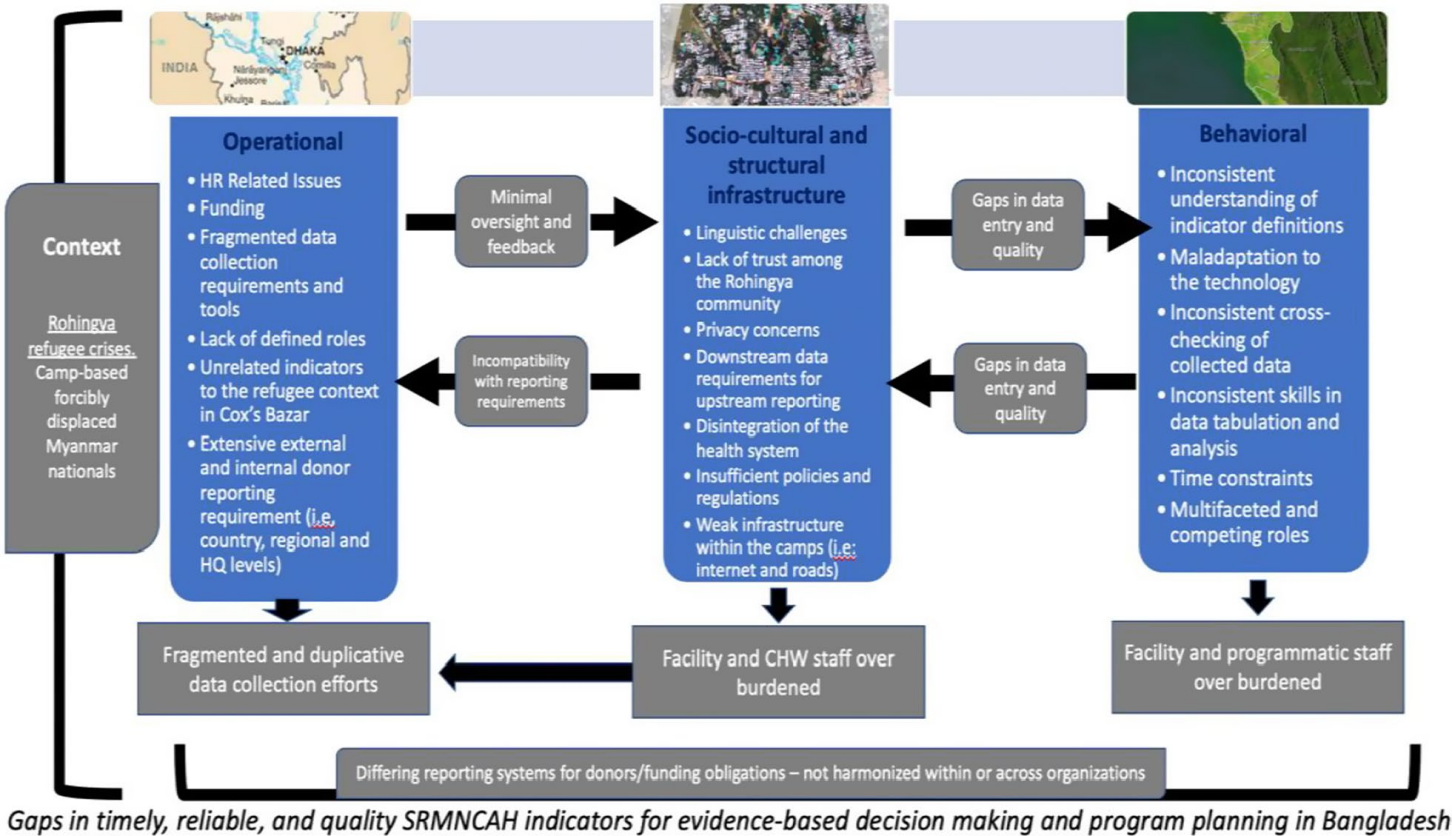
Perceived challenges and gaps in current systems and resources for collection of SRMNCAH indicators, reported by our stakeholders

The study findings also indicate gaps in resources and systems, including internal capacity, funding, and materials. Irrespective of access to resources, challenges with infrastructure at the ground level impedes the quality of upstream data distribution and analysis. Due to the scarcity of resources, the abundance of reporting systems, and a lack of national buy-in, organizations in camp-based settings are required to input and analyze their data manually. Therefore, it is difficult for frontline staff to report their indicators in an accurate and timely manner, negatively impacting the overall quality of data (See Fig. 3).

### Ethical considerations

Study participants complained from the discordance between their expected labor outputs for national and donor reporting requirements, dearth of funding and investment in human resources and the impact on data confidentiality and privacy. One Health Information System manager (KII participant 5) explained,

“*People don’t have robust HIS systems. People don’t have the capacity to create something like that in terms of financial or human resource capacity. Also, the focus is not there, this is definitely underfunded ... If people also don’t have the literacy regarding the data, can they use this for evidence creation? How can this evidence actually help them improve their programs? People really need to learn how to maintain confidential data, how to use confidentiality of data, and how to omit them. They need to learn everything*.”

The facility assessment confirms that primary health clinics are significantly understaffed and have insufficient privacy and confidentiality measures set in place. Indeed, a number of the facilities visited did not have an allocated position for data entry or data management. As a result, the responsibility of data entry often fell to the nurse, doctor and/or midwife on site.

Finally, the findings indicate that traditional reporting systems are incompatible with the socio-cultural norms of the Rohingya population. Indeed, many of the KII and FGD participants discussed the pervasive mistrust of the Rohingya population in aid and government services and their inability to identify patients. As thoroughly explained by a community health worker (KII participant 6), “*The challenge in the camp is that we do not have any way to identify a unique patient. Let's say a woman comes to a health facility without a national identification card. Here [in the camp], we still do not have any health card as of now, like a health card by which we can say that this woman has received this number of services, she’s come this time—you know? Tracking that patient overall is not possible*.”

As a result, gaps in representative and population-level data and identification of SRMNCAH priorities continue to persist. A program officer from a national NGO further explained (KII participant 7),

“*…….we can’t collect the population level data. There are challenges. If somebody is coming for the ANC consultation and the same person is going for family planning, we know this is the same person. But for the family planning consultation, we have no way to track the patient. So, you have to manually count each patient*.”

## Discussion

The findings from this multi-method feasibility assessment provide a comprehensive overview of the feasibility of collecting a core set of SRMNCAH indicators in the humanitarian context of Bangladesh for improved humanitarian response. The study results note overall support and enthusiasm for developing a core set of SRMNCAH indicators among the different humanitarian stakeholders in Cox’s Bazar. Aligned with findings from other studies conducted in Bangladesh [22, 23], representatives from a variety of institutions highlighted numerous existing resources and systems that could be leveraged and improved to ensure the feasibility of collecting this core set of indicators for monitoring and evaluating SRMNCAH health services and outcomes in the humanitarian settings in Cox’s Bazar. The study findings, further, indicate that a core set of facility-based and community-level indicators compared to population-level indicators were perceived to be more relevant and feasible given the current context in Cox’s Bazar. Further, participants overarchingly agreed that population-based indicators are not feasible to collect due to a lack of patient health history and treatment tracking systems among FDMNs residing in the camps in Cox’s Bazar expressed their concerns with privacy and confidentiality of the data. Indeed, studies conducted by Begum et al. and Kiberu et al., found that increased data ownership among the community is crucial to address the community’s concerns surrounding privacy and confidentiality of their data and in turn improve overarching data quality and reporting [17, 24].

The study findings also indicate that the current status of requested SRMNCAH data collection from donors, international and United Nations (UN) agencies, coordination/cluster systems into different reporting systems is fragmented and especially burdensome at the facility and community level. Barriers to quality, reliable and timely SRMNCAH data reported by the study respondents, are often similar to those reported in other developing nations [17, 23–25], including inadequate, (1) human resources; (2) compatible infrastructure and reporting systems and requirements (i.e., low internet connectivity, power outages, scarcity of technology); (3 training and capacity; (4) sociopolitical barriers; (5) privacy; and (6) confidentiality and (7) funding. A mixed-methods assessment of routine health information systems and data conducted in Addis Ababa found that the extensive presence of parallel reporting and unstandardized routine data collection practices resulted in over and under reporting of health indicators and had negative implications on service provider’s perceptions toward routine health data collection practice, impacting data completeness and quality [25].

This is further complicated by in the context of Bangladesh, where SRMNCAH services are offered through a two-tiered system with siloed HMIS systems,^5^ under the Ministry of Health and Family Welfare (MOHFW): the Directorate General of Health Services (DGHS) and the Directorate General of Family Planning (DGFP) [14]. Bangladesh has adopted and implemented a harmonized, nationally endorsed reporting system District Health Information Software 2 (DHIS2) to collect an array of health indicators [18, 26]. With Bangladesh’s HMIS considered as an active contributor to the global DHIS2 implementation strategy and concerted national efforts to improve overarching health data reporting, it is not surprising the data reporting rate through DHIS2 was 98% [26, 27]. However, reporting data quality and timeliness has been reported as poor in both the literature [17, 26] and by the study participants. The findings, supported by the literature, indicates that as a result of delayed and poor data quality and reporting, policy makers, government, and NGO programme developers rely on periodic surveys instead of the DHIS2 data [26]. Further, there is great variety in data collection information systems that international and national partner organizations develop, implement, and monitor service access by forcibly displaced Myanmar nationals (FDMN) in Cox’s Bazar. However, systems used by international organizations are distinct from those used by DGHS and DGFP in Bangladesh. Indisputably, multi-year- multi-sector interventions would be required to increase the feasibility for countries facing humanitarian and emergency contexts to collect a core set of SRMNCAH quality indicators.

The results also highlighted that leveraging available resources, coupled with a number of macro, programmatic and training and resource recommendations should be taken into consideration. At the macro-level, national policies and regulations surrounding civil registration need to be amended to enhance data collection practices, as the lack of a civil registration system/policies for forcibly displaced Myanmar nationals (FDMN) complicates registration for refugees residing in the camps (e.g., birth registration, child registration) and prevents health facilities to have access to the needed registration information. Further, national policies and regulations surrounding the HIV/AIDS, prohibits providing services at the camp-based health facilities and thus, collecting HIV/AIDS related indicators are not feasible there.

The findings as well indicated the need to consider the inclusion of additional indicators related to capture: coordination measures, child violence, early marriage, further age disaggregation (maternal and SGBV indicators), immunization and community health workers (CHW) outreach are warranted given the SRMNCAH context in Cox’s Bazar. The findings also suggested that including indicators related to coordination measures can help address gaps in referral pathways while also promoting accountability among implementing agencies [28]; coordination indicators can also provide agencies with an understanding of capacity and service availability within a geographical area to balance distribution and duplication of labor. As a result of displacement, early marriage is highly prevalent among the Rohingya community [29], therefore cross-cutting indicators surrounding early marriage, maternal and newborn health should be considered. Stakeholders also noted the need to evaluate community awareness of service availability by CHW and health education of the community, and therefore including indicators surrounding activities implemented at the community level is warranted.

An array of training is necessary before the rollout of the proposed list of indicators. First and foremost, solid resources and training should be provided for staff members surrounding data security, storage and privacy. The goal here is to protect the patient while simultaneously meeting global standards for data privacy, particularly in regard to indicators that are considered socio-cultural taboos. Training on the new Inter-Agency Field Manual on Reproductive Health in Humanitarian settings [29] in conjunction with values clarification and attitude transformation workshops would be beneficial for health workers tasked with reporting on these indicators and working on sexual and reproductive health. Some of the indicators’-specific training required could include: training on different contraception modalities that can be used for emergency contraception (EC), training on how to report stillbirths, training of staff and creating and distributing manuals on types of maternal and newborn morbidities, and materials and training on a wide range of STIs.

## Strengths and limitations

We used Guba’s [30] recommended strategies, along with assessments of credibility, confirmability and transferability to evaluate the trustworthiness of our findings. For example, we established data credibility through regular debriefs with both teams in Bangladesh and Canada, triangulation in assessment, check-ins with members and prolonged engagement with the field. Furthermore, we were able to incorporate data from various sources using our multi-methods study design. Indeed, the qualitative data in our study allowed us to explore quantitative findings, with facility assessments helping to identify key themes from the FGDs and KIIs, as well as informing on available SRMNCAH resources in addition to current monitoring and evaluation systems. However, our study also presents several limitations. For instance, we faced difficulties in accessing and locating some SRMNCAH monitoring and evaluation records, which led to challenges in documentation. In addition, language barriers may have caused gaps in understanding between the participants and members of the research team, as some participant responses were simultaneously translated using an interpreter during the interview. Finally, it is possible that researcher bias may have affected participant-researcher interaction and the interpretation of data. To counter these limitations, our team used memoing and regular debriefings, which helped us critically assess these dynamics and therefore improve the trustworthiness of the data.

## Conclusion

The findings from this multi-methods feasibility assessment suggested that there is a widespread support and enthusiasm for developing a core set of sexual reproductive maternal newborn child and adolescent health (SRMNCAH) indicators among the different humanitarian stakeholders in Cox’s Bazar. The current status of requested data collection from donors, the national government, international and United Nations (UN) agencies, coordination/cluster systems into different reporting systems is highly variable and burdensome. The proposed indicators’ list should be accompanied with a toolkit to be tailored and distributed to provincial, health facility and community staff members to facilitate data collection by explaining the link between reporting and program improvement to enhance buy-in across staff members tasked with data collection. The training, policy changes, and resources described above would work to ensure accurate and quality data collection across Bangladesh.

## Data Availability

Available upon request.

## Abbreviations

ANC: Antenatal care
CAC: Comprehensive abortion care
CHW: Community health worker
DGFP: Directorate General of Family Planning
DGHS: Directorate General of Health Services
DHIS2: District Health Information System 2
EWARS: Early Warning and Reporting System
FDMN: Forcibly Displaced Myanmar Nationals
FGD: Focus group discussion
GBV: Gender-based violence
GDPR: General data protection regulation
HIS: Health Information System
HMIS: Health Management Information System
KI: Key informant
KII: Key informant interview
MoH: Ministry of Health
MoHFW: Ministry of Health and Family Welfare
NGO: Non-Governmental Organization
PMTCT: Prevention of mother to child transmission
RTI: Reproductive tract infection
SGBV: Sexual and gender-based violence
STI: Sexually transmitted infection
SRH: Sexual and reproductive health
SRMNCAH: Sexual, reproductive, maternal, neonatal, child and adolescent health
UNICEF: United Nations Children’s Fund
UN: United Nations
WHO: World Health Organization

## Footnotes

1 The government of Bangladesh doesn’t recognize Rohingya people as refugees, and therefore are referenced as forcibly displaced Myanmar nationals throughout this report.

2 The health system goes through a period of degradation and fragmentation due to increasing violence and insecurity, weakening governance, and loss of resources [9].

3 A more detailed description of this projects’ methodology can be found in the report for the WHO.

4 Menstrual regulation (MR) is a legal procedure that uses manual vacuum aspiration or a combination of mifepristone and misoprostol to regulate the menstrual cycle when menstruation is absent for a short duration. While abortion is illegal in Bangladesh, MR allows for safe pregnancy termination.

5 The two directorates use different HMIS and only DGHS uses DHIS2.
